# Supplementary material for: The Evolution and Characterization of the RNA Interference Pathways in Lophotrochozoa
Source: Genome Biol Evol. 2024 May 7;16(5):evae098. doi: 10.1093/gbe/evae098 (PMC11114477; doi:10.1093/gbe/evae098)
Supplement: evae098_Supplementary_Data [file evae098_supplementary_data.zip › Supplementary_materials.zip!/selected_miRNAs/NoGe_selected_miRNAs/mirdeep_pdf_files/NMRB01001254.1_Notospermus_geniculatus_strain_Ushimado_scaffold1254,_whole_genome_shotgun_sequence_26724.pdf]

Provisional ID	: NMRB01001254.1_Notospermus_geniculatus_strain_Ushimado_scaffold1254,_whole_genome_shotgun_sequence_26724
Score total	: 113444.2
Score for star read(s)	: 3.9
Score for read counts	: 113435.7
Score for mfe	: 1.6
Score for randfold	: 0
Score for cons. seed	: 3
Total read count	: 222511
Mature read count	: 222441
Loop read count	: 0
Star read count	: 70

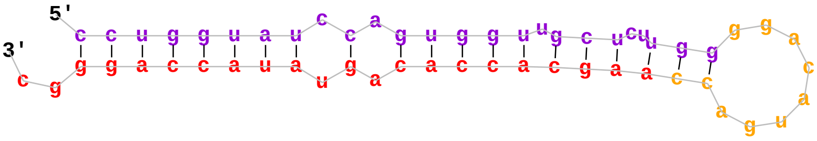

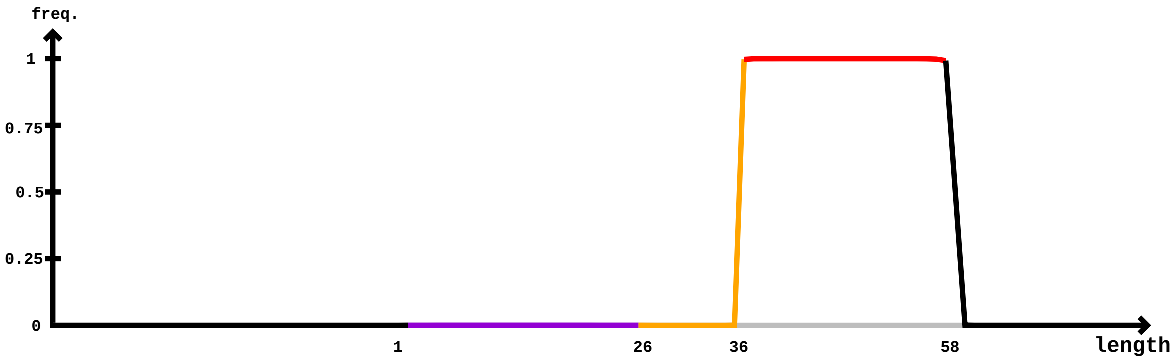

Star Mature

```
5'- uaucagauaauggagaaaauugcauggcuuugucc ccugguauccagugguugcucuugg ggacaugacc aagcaccacaguauaccagggc auaugcaugcuucuagauuc   -3'   obs
    uaucagauaauggagaaaauugcauggcuuuguccccugguauccagugguugcucuuggggacaugaccaagcaccacaguauaccagggcauaugcaugcuucuagauuc         exp
    .....((...(((((......(((((....(((.(((((((((.(.(((((.(((..(((........))))))))))).).))))))))).)))..))))))))))...))     reads   mm   sample
    ...................................ccugguauccagugguugcucu.......................................................     1       0    Mm3
    ...................................ccugguauccagugguugcucuug.....................................................     1       0    Mm3
    ...................................ccugguaAccagugguugcucuugg....................................................     1       1    Mm3
    ...................................ccugguauccagugguugcucuugg....................................................     9       0    Mm3
    ...................................ccugguauccagugguugcucuuggA...................................................     3       1    Mm3
    ...................................ccugguauccagugguugcucuuggggacaugaccU.........................................     1       1    Mm3
    .....................................................................caagcaccacaguauaccaggg.....................     3       0    Mm3
    .....................................................................caagcaccacaguauaccagggc....................     16      0    Mm3
    .....................................................................caagcaccacaguauaccagggcU...................     1       1    Mm3
    .....................................................................caagcaccacaguauaccagggca...................     1       0    Mm3
    ......................................................................aagcaccacaguauaccag.......................     2       0    Mm3
    ......................................................................aagcaccacaguauaccUg.......................     1       1    Mm3
    ......................................................................aagcaccacaguauaccagg......................     30      0    Mm3
    ......................................................................aagcaccacaguauaccCggg.....................     2       1    Mm3
    ......................................................................aagcaccacaguUuaccaggg.....................     1       1    Mm3
    ......................................................................aagcaccacaguauaccaggA.....................     2       1    Mm3
    ......................................................................aagcaccacaguauaccGggg.....................     1       1    Mm3
    ......................................................................aagcaccacaguauacAaggg.....................     1       1    Mm3
    ......................................................................aagcaccacaguauaccaggC.....................     2       1    Mm3
    ......................................................................aagcaccacaguauaccaggg.....................     159     0    Mm3
    ......................................................................aagcaccacaguauaccaggU.....................     1       1    Mm3
    ......................................................................aagcacAacaguauaccagggc....................     1       1    Mm3
    ......................................................................aagcaccacaguauacAagggc....................     145     1    Mm3
    ......................................................................aagcaccacaguauaccaCggc....................     1       1    Mm3
    ......................................................................aagcaccacaguauaccagUgc....................     6       1    Mm3
    ......................................................................aagcaccacaguUuaccagggc....................     5       1    Mm3
    ......................................................................aUgcaccacaguauaccagggc....................     4       1    Mm3
    ......................................................................aagcUccacaguauaccagggc....................     7       1    Mm3
    ......................................................................aagcaccacaguauaccagggA....................     22      1    Mm3
    ......................................................................aagcaGcacaguauaccagggc....................     2       1    Mm3
    ......................................................................aagcaUcacaguauaccagggc....................     6       1    Mm3
    ......................................................................aagcaccacaguauaccUgggc....................     94      1    Mm3
    ......................................................................aagcaccacaCuauaccagggc....................     35      1    Mm3
```

Star **Mature**

uaucagauaauggagaaaauugcauggcuuugucc**ccugguauccagugguugcucuugg**ggacaugacc**aagcaccacaguauaccagggc**auaugcaugcuucuagauuc			
....aagcaccacagCauaccagggc....................	4	1	Mm3
....aagGaccacaguauaccagggc....................	2	1	Mm3
....aaUcaccacaguauaccagggc....................	5	1	Mm3
....aagcGccacaguauaccagggc....................	3	1	Mm3
....aagcaccacGguauaccagggc....................	23	1	Mm3
....aagcaccacaguauaccaggAc....................	5	1	Mm3
....aCgcaccacaguauaccagggc....................	3	1	Mm3
....aagcaccacaguGuaccagggc....................	4	1	Mm3
....aagUaccacaguauaccagggc....................	5	1	Mm3
....aagcaccacagAauaccagggc....................	2	1	Mm3
....aagcaccacaAuauaccagggc....................	40	1	Mm3
....aagcaccacaguauaccaUggc....................	4	1	Mm3
....Cagcaccacaguauaccagggc....................	2	1	Mm3
....aagcaNcacaguauaccagggc....................	4	1	Mm3
....aagcaccacaguauaccaAggc....................	3	1	Mm3
....aagcacNacaguauaccagggc....................	3	1	Mm3
....aagcaccacaguauaccCgggc....................	107	1	Mm3
....aagcaccacCguauaccagggc....................	1	1	Mm3
....aagcaccacaguauaccagggU....................	55	1	Mm3
....Uagcaccacaguauaccagggc....................	14	1	Mm3
....aagcaAcacaguauaccagggc....................	2	1	Mm3
....aagcaccGcaguauaccagggc....................	5	1	Mm3
....aagcaccacaguauaUcagggc....................	4	1	Mm3
....aagcaccacaguauaccGgggc....................	71	1	Mm3
....aagcaccacaguauaccagCgc....................	1	1	Mm3
....aagcCccacaguauaccagggc....................	3	1	Mm3
....aGgcaccacaguauaccagggc....................	6	1	Mm3
....aagcaccacaguauaccagggc....................	29157	0	Mm3
....aagcaccUcaguauaccagggc....................	6	1	Mm3
....aagcaccacaguauacUagggc....................	97	1	Mm3
....aagcaccacaguauaccagggG....................	4	1	Mm3
....aagcacUacaguauaccagggc....................	15	1	Mm3
....Nagcaccacaguauaccagggc....................	18	1	Mm3
....aagcaccacaguauGccagggc....................	13	1	Mm3
....aagcaccacaguauCccagggc....................	1	1	Mm3
....aaCcaccacaguauaccagggc....................	1	1	Mm3
....aagcaccacaguaCaccagggc....................	4	1	Mm3
....aagcaccacaguauacGagggc....................	37	1	Mm3
....aagcaccacaguauaccaggCc....................	15	1	Mm3
....aagcaccaUaguauaccagggc....................	16	1	Mm3
....aagcaccacaguauaccaggUc....................	7	1	Mm3
....aagcaccacaUuauaccagggc....................	20	1	Mm3
....aagcaccacaguauUccagggc....................	3	1	Mm3
....aagcaccacUguauaccagggc....................	4	1	Mm3
....Gagcaccacaguauaccagggc....................	8	1	Mm3
....aagcaccacaguauaccagAgc....................	8	1	Mm3
....aaAcaccacaguauaccagggc....................	7	1	Mm3
....aagcaccCcaguauaccagggc....................	5	1	Mm3
....aagcaccacaguaAaccagggc....................	4	1	Mm3
....aagcaccacaguaGaccagggc....................	3	1	Mm3
....aagcaccacaguauaccagggcU...................	528	1	Mm3
....aagcaccacaguauaccagggcG...................	9	1	Mm3
....aagcaccacaguauaccagggca...................	54	0	Mm3
....aagcaccacaguauaccagggcC...................	14	1	Mm3
....aagcaccacaguauaccagggcau..................	4	0	Mm3
....aagcaccacaguauaccagggcaA..................	1	1	Mm3
....aagcaccaGaguauaccagggcau..................	4	1	Mm3
....aagcaccacaguauaccagggcaG..................	1	1	Mm3
....aagcaccacaguauaccagggcCu..................	3	1	Mm3
....aagcaccacaguauaccagggcUu..................	14	1	Mm3
....aagcaccacaguauaccagggcaAa.................	1	1	Mm3
....aagcaccacaguauaccagggcauU.................	1	1	Mm3
....aagcaccaGaguauaccagggcaua.................	1	1	Mm3
....aUcaccacaguauaccagggc....................	1	1	Mm3
....agcaccacaguauaccagggc....................	67	0	Mm3
....agcaccacCguauaccagggc....................	1	1	Mm3
....agcacAacaguauaccagggc....................	1	1	Mm3
....agGaccacaguauaccagggc....................	1	1	Mm3
....agcaccacaguauaccagUgc....................	1	1	Mm3
....agcaccacaguauaccagggca...................	2	0	Mm3

Star Mature

uaucagauaauggagaaaauugcauggcuuugucc**ccugguauccagugguugcucuugg**ggacaugacc**aagcaccacaguauaccagggc**auaugcaugcuucuagauuc			
....agcaccacaguauaccagggcU...................	5	1	Mm3
....gcaccacaguauaccagggc....................	6	0	Mm3
....gcaccacaguauaccagggcU...................	1	1	Mm3
....caccacaguauaccagggc....................	6	0	Mm3
....caccacaguauaccagggcU...................	1	1	Mm3
....accacaguauaccagggc....................	14	0	Mm3
...................................ccugguauccagugguugcucuugg..	2	0	Fm3
....gaUcaagcaccacaguauaccagggc....................	2	1	Fm3
....caagcaccacaguauaccaggg.....................	4	0	Fm3
....caagcaccacaguauaccagggc....................	18	0	Fm3
....Uaagcaccacaguauaccagggc....................	2	1	Fm3
....caagcaccacaguGuaccagggc....................	1	1	Fm3
....aagcaccacaguauaccGg.......................	1	1	Fm3
....aagcaccacaguauaccag.......................	63	0	Fm3
....aagcacUacaguauaccag.......................	1	1	Fm3
....aagcaccacaguauaccagU......................	6	1	Fm3
....aagcaccacaguauacAagg......................	2	1	Fm3
....Uagcaccacaguauaccagg......................	1	1	Fm3
....aagcaccacaguauaccagg......................	151	0	Fm3
....aagcaccacaguauacUagg......................	1	1	Fm3
....aagcaccacaguauaccUggg.....................	2	1	Fm3
....Nagcaccacaguauaccaggg.....................	1	1	Fm3
....aagcaccacaguauacAaggg.....................	1	1	Fm3
....aagcaccacaguauacUaggg.....................	1	1	Fm3
....aagcaccacaguauaccaggU.....................	7	1	Fm3
....aagcacGacaguauaccaggg.....................	1	1	Fm3
....aagcaccacaguauacGaggg.....................	2	1	Fm3
....aagcaccacaguauaccGggg.....................	3	1	Fm3
....aagcaccacaguauaccaggC.....................	3	1	Fm3
....aagcaccacaguauaccaggg.....................	521	0	Fm3
....aagcaccacaAuauaccagggc....................	55	1	Fm3
....aagcaccacaguauaccagggG....................	2	1	Fm3
....aagcaccacaguauacAagggc....................	182	1	Fm3
....aagcaccacaUuauaccagggc....................	30	1	Fm3
....aagcaccacaguauaccagCgc....................	2	1	Fm3
....Nagcaccacaguauaccagggc....................	23	1	Fm3
....aagcaccacaguauacGagggc....................	52	1	Fm3
....aagcaccacaguauaccagggc....................	37490	0	Fm3
....aagcaccacaguauaccUgggc....................	118	1	Fm3
....Uagcaccacaguauaccagggc....................	28	1	Fm3
....aagGaccacaguauaccagggc....................	7	1	Fm3
....aagAaccacaguauaccagggc....................	8	1	Fm3
....aagcaccacagAauaccagggc....................	5	1	Fm3
....aagcaccCcaguauaccagggc....................	1	1	Fm3
....aagcaccacaguauaccagUgc....................	11	1	Fm3
....aagcaccaUaguauaccagggc....................	33	1	Fm3
....aUgcaccacaguauaccagggc....................	4	1	Fm3
....aagcaccacaguauaccaUggc....................	21	1	Fm3
....aagcaccacagGauaccagggc....................	2	1	Fm3
....aagcaccacaguauaccagggU....................	85	1	Fm3
....aagcaccacaguauaAcagggc....................	2	1	Fm3
....aaCcaccacaguauaccagggc....................	5	1	Fm3
....aagcaccacaguauaccaggCc....................	6	1	Fm3
....aagcacNacaguauaccagggc....................	5	1	Fm3
....aaUcaccacaguauaccagggc....................	6	1	Fm3
....aagcaAcacaguauaccagggc....................	1	1	Fm3
....aagcaccacaguauaccGgggc....................	117	1	Fm3
....aagcacAacaguauaccagggc....................	3	1	Fm3
....aagcaccacaguauUccagggc....................	2	1	Fm3
....aGgcaccacaguauaccagggc....................	6	1	Fm3
....aagcacUacaguauaccagggc....................	19	1	Fm3
....aagcaccaAaguauaccagggc....................	3	1	Fm3
....Gagcaccacaguauaccagggc....................	6	1	Fm3
....aagcaccacaguauaccagggA....................	42	1	Fm3
....aCgcaccacaguauaccagggc....................	5	1	Fm3
....aagcaUcacaguauaccagggc....................	11	1	Fm3
....aagcaccacaguaCaccagggc....................	6	1	Fm3
....aagcaccacaguauaUcagggc....................	14	1	Fm3
....aagcGccacaguauaccagggc....................	4	1	Fm3

```
                                   Star                              Mature
uaucagauaauggagaaaauugcauggcuuuguccccugguauccagugguugcucuuggggacaugaccaagcaccacaguauaccagggcauaugcaugcuucuagauuc
......................................................................aagcaccacaguauaccaggAc....................    4     1    Fm3
......................................................................aagcUccacaguauaccagggc....................    3     1    Fm3
......................................................................aagcaGcacaguauaccagggc....................    3     1    Fm3
......................................................................aagcaccacaguUuaccagggc....................    4     1    Fm3
......................................................................aagcacGacaguauaccagggc....................    6     1    Fm3
......................................................................aagcaccacGguauaccagggc....................    29    1    Fm3
......................................................................aagcaccacaguauacUagggc....................    137   1    Fm3
......................................................................aagcaccacaCuauaccagggc....................    37    1    Fm3
......................................................................aagUaccacaguauaccagggc....................    4     1    Fm3
......................................................................aagcaccacaguGuaccagggc....................    2     1    Fm3
......................................................................aagcaccacaguauaccaAggc....................    2     1    Fm3
......................................................................aaAcaccacaguauaccagggc....................    9     1    Fm3
......................................................................aagcaccacaguauaccagAgc....................    6     1    Fm3
......................................................................aagcaccacUguauaccagggc....................    5     1    Fm3
......................................................................aagcaccacCguauaccagggc....................    1     1    Fm3
......................................................................aagcaccacaguCuaccagggc....................    2     1    Fm3
......................................................................aagcaccGcaguauaccagggc....................    10    1    Fm3
......................................................................aagcaccUcaguauaccagggc....................    10    1    Fm3
......................................................................aagcaccacaguauaccaggUc....................    8     1    Fm3
......................................................................aagcaccacagCauaccagggc....................    5     1    Fm3
......................................................................aagcCccacaguauaccagggc....................    5     1    Fm3
......................................................................aagcaccacaguaGaccagggc....................    4     1    Fm3
......................................................................aagcaccacaguauGccagggc....................    7     1    Fm3
......................................................................aagcaccacaguauaccCgggc....................    116   1    Fm3
......................................................................aagcaNcacaguauaccagggc....................    9     1    Fm3
......................................................................aagcaccacaguaAaccagggc....................    6     1    Fm3
......................................................................aagcaccacaguauaccagggca...................    105   0    Fm3
......................................................................aagcaccacaguauaccagggcU...................    224   1    Fm3
......................................................................aagcaccaUaguauaccagggca...................    8     1    Fm3
......................................................................aagcaccacaguauaccagggcC...................    3     1    Fm3
......................................................................aagcaccacaguauacUagggca...................    2     1    Fm3
......................................................................aagcaccacaguauaccagggUau..................    1     1    Fm3
......................................................................aagcaccacaguauaccagggcau..................    6     0    Fm3
......................................................................aagcaccaGaguauaccagggcau..................    6     1    Fm3
.......................................................................agcaccacaguauaccaAggc....................    1     1    Fm3
.......................................................................agcaccacaguauaccagggc....................    109   0    Fm3
.......................................................................agcaccacagAauaccagggc....................    1     1    Fm3
.......................................................................agcaccacaguauaccaCggc....................    1     1    Fm3
.......................................................................agcaccacagCauaccagggc....................    1     1    Fm3
.......................................................................agcaccacaguauaccCgggc....................    1     1    Fm3
.......................................................................agcaccacagGauaccagggc....................    1     1    Fm3
.......................................................................agcaccacaguauaccagUgc....................    1     1    Fm3
.......................................................................agcaccacaguauaccaggUc....................    1     1    Fm3
.......................................................................aUcaccacaguauaccagggc....................    3     1    Fm3
.......................................................................agcaccacaguauaccagggcU...................    1     1    Fm3
........................................................................gcaccacaguauaccagggc....................    1     0    Fm3
.........................................................................caccacaguauaccagggc....................    1     0    Fm3
..........................................................................accacaguauaccagggc....................    11    0    Fm3
...........................................................................ccacaguauaccagggcU...................    1     1    Fm3

...................................ccugguauccagugguugcu.........................................................    1     0    Mm2
...................................ccugguauccagugguugcucuu......................................................    1     0    Mm2
...................................ccugguauccagugguugcucuug.....................................................    5     0    Mm2
...................................ccugguauccagugguGgcucuug.....................................................    1     1    Mm2
...................................ccugguauccagugguCgcucuugg....................................................    1     1    Mm2
...................................ccugguauccagugguugcucuugg....................................................    22    0    Mm2
....................................cugguauccagugguugcucuugg....................................................    1     0    Mm2
......................................gguauccagugguugcucuugg....................................................    1     0    Mm2
.....................................................................caagcaccacaguauaccaggg.....................    1     0    Mm2
.....................................................................caagcaccacaguauaGcagggc....................    1     1    Mm2
.....................................................................caagcaccacaguauacAagggc....................    3     1    Mm2
.....................................................................Aaagcaccacaguauaccagggc....................    2     1    Mm2
.....................................................................caagcaccacaguauacGagggc....................    1     1    Mm2
.....................................................................caagcaccacaguauaccagggc....................    51    0    Mm2
.....................................................................caagcaccacaguauaccagggcU...................    2     1    Mm2
......................................................................aagcaccacaguauaccag.......................    7     0    Mm2
......................................................................aagcaccacaguauaccagg......................    46    0    Mm2
......................................................................aagcaccacaguauaccGgg......................    1     1    Mm2
......................................................................aagcaccacaguauaccaggC.....................    7     1    Mm2
......................................................................aagcaccacGguauaccaggg.....................    1     1    Mm2
```

```
                                   Star                                Mature
uaucagauaauggagaaaauugcauggcuuugucccugguauccagugguugcucuggggacaugaccaagcaccacaguauaccagggcauaugcaugcuucuagauuc
.....................................................................Uagcaccacaguauaccaggg.....................    1    1    Mm2
.....................................................................aagcaccacaguauacAaggg.....................    1    1    Mm2
.....................................................................aagcaccacaguauaccGggg.....................    1    1    Mm2
.....................................................................aagcaccacaguauaccCggg.....................    3    1    Mm2
.....................................................................aagcaccacaAuauaccaggg.....................    1    1    Mm2
.....................................................................aagcaccacaguauaccaggg.....................    280    0    Mm2
.....................................................................aagcUccacaguauaccaggg.....................    1    1    Mm2
.....................................................................aagcaccacaCuauaccaggg.....................    1    1    Mm2
.....................................................................aagcaccacaguaCaccagggc....................    14    1    Mm2
.....................................................................aagcUccacaguauaccagggc....................    16    1    Mm2
.....................................................................aagcaccacagCauaccagggc....................    8    1    Mm2
.....................................................................aagcaccacaguauGccagggc....................    29    1    Mm2
.....................................................................aagcCccacaguauaccagggc....................    6    1    Mm2
.....................................................................aagcaccacaguauCccagggc....................    4    1    Mm2
.....................................................................aagcaccUcaguauaccagggc....................    21    1    Mm2
.....................................................................aagcaccacaguauacGagggc....................    154    1    Mm2
.....................................................................aagcaGcacaguauaccagggc....................    3    1    Mm2
.....................................................................aagcaNcacaguauaccagggc....................    11    1    Mm2
.....................................................................aagcaccCcaguauaccagggc....................    7    1    Mm2
.....................................................................aagcaccacaguauaccCgggc....................    306    1    Mm2
.....................................................................aagcaccacaguauaccaAggc....................    7    1    Mm2
.....................................................................aagcacNacaguauaccagggc....................    7    1    Mm2
.....................................................................aagcaccacaguaGaccagggc....................    9    1    Mm2
.....................................................................aagcGccacaguauaccagggc....................    17    1    Mm2
.....................................................................aagcaccacUguauaccagggc....................    13    1    Mm2
.....................................................................aagcaccacaguauaccaCggc....................    6    1    Mm2
.....................................................................aagcacAacaguauaccagggc....................    4    1    Mm2
.....................................................................aagcaccacaguaAaccagggc....................    15    1    Mm2
.....................................................................Uagcaccacaguauaccagggc....................    79    1    Mm2
.....................................................................aagcaccacaguauaccGgggc....................    301    1    Mm2
.....................................................................aagcaccacaguauaccagCgc....................    7    1    Mm2
.....................................................................Gagcaccacaguauaccagggc....................    23    1    Mm2
.....................................................................aUgcaccacaguauaccagggc....................    14    1    Mm2
.....................................................................aaCcaccacaguauaccagggc....................    9    1    Mm2
.....................................................................aagGaccacaguauaccagggc....................    7    1    Mm2
.....................................................................aagcaccacaguauaccaggCc....................    17    1    Mm2
.....................................................................aagcaccacaguauaGcagggc....................    2    1    Mm2
.....................................................................aaAcaccacaguauaccagggc....................    19    1    Mm2
.....................................................................aagcaccacaguauaccagggA....................    18    1    Mm2
.....................................................................aagcaccacaguauaccagUgc....................    18    1    Mm2
.....................................................................aagcaccacaguauacAagggc....................    500    1    Mm2
.....................................................................aGgcaccacaguauaccagggc....................    20    1    Mm2
.....................................................................aagcaccacaguauaccaggUc....................    20    1    Mm2
.....................................................................aagcaccacaguauacUagggc....................    332    1    Mm2
.....................................................................aagcaccacaguauaccagggG....................    14    1    Mm2
.....................................................................aagcaccacaCuauaccagggc....................    88    1    Mm2
.....................................................................aagcaccacaguauaUcagggc....................    14    1    Mm2
.....................................................................aagcaccaUaguauaccagggc....................    43    1    Mm2
.....................................................................aagcaccacaguUuaccagggc....................    4    1    Mm2
.....................................................................aagcacUacaguauaccagggc....................    43    1    Mm2
.....................................................................Cagcaccacaguauaccagggc....................    7    1    Mm2
.....................................................................aagcaccacaguauaccaUggc....................    14    1    Mm2
.....................................................................aagcaccacCguauaccagggc....................    4    1    Mm2
.....................................................................aagcacGacaguauaccagggc....................    3    1    Mm2
.....................................................................Nagcaccacaguauaccagggc....................    59    1    Mm2
.....................................................................aagcaccacaguauaccagAgc....................    15    1    Mm2
.....................................................................aagAaccacaguauaccagggc....................    19    1    Mm2
.....................................................................aagcaccacaguauaccagggc....................    103434    0    Mm2
.....................................................................aagcaccacagAauaccagggc....................    9    1    Mm2
.....................................................................aagcaccacaUuauaccagggc....................    64    1    Mm2
.....................................................................aagcaccacaguauaccagggU....................    134    1    Mm2
.....................................................................aagcaccacaguGuaccagggc....................    10    1    Mm2
.....................................................................aagcaUcacaguauaccagggc....................    14    1    Mm2
.....................................................................aagcaccacaguauUccagggc....................    14    1    Mm2
.....................................................................aagcaccacaguauaccUgggc....................    285    1    Mm2
.....................................................................aagcaccaAaguauaccagggc....................    9    1    Mm2
.....................................................................aaNcaccacaguauaccagggc....................    4    1    Mm2
.....................................................................aagcaccacaguauaccaggAc....................    19    1    Mm2
.....................................................................aagcaccacagGauaccagggc....................    10    1    Mm2
.....................................................................aagcaccacGguauaccagggc....................    59    1    Mm2
```

```
                                   Star                                Mature
uaucagauaauggagaaaauugcauggcuuuguccccugguauccagugguugcucuuggggacaugaccaagcaccacaguauaccagggcauaugcaugcuucuagauuc
......................................................................aagcaccacaAuauaccagggc....................   154   1   Mm2
......................................................................aaUcaccacaguauaccagggc....................   27    1   Mm2
......................................................................aagUaccacaguauaccagggc....................   12    1   Mm2
......................................................................aCgcaccacaguauaccagggc....................   7     1   Mm2
......................................................................aagcaccacaguauaAcagggc....................   6     1   Mm2
......................................................................aagcaAcacaguauaccagggc....................   2     1   Mm2
......................................................................aagcaccacaguCuaccagggc....................   4     1   Mm2
......................................................................aagcaccGcaguauaccagggc....................   12    1   Mm2
......................................................................aagcaccacaguauaccagggcC...................   17    1   Mm2
......................................................................aagcaccacaguauaccUgggca...................   1     1   Mm2
......................................................................aagcaccacaguauacUagggca...................   1     1   Mm2
......................................................................aagcacUacaguauaccagggca...................   1     1   Mm2
......................................................................aagcaccacaguauaccagggca...................   110   0   Mm2
......................................................................aagcaccacaguauaccagggcG...................   14    1   Mm2
......................................................................aagcaccacaguauaccagggcU...................   809   1   Mm2
......................................................................aagcaccaGaguauaccagggcau..................   20    1   Mm2
......................................................................aagcaccacaguauaccagggcCu..................   6     1   Mm2
......................................................................aagcaccacaguauaccagggcaA..................   2     1   Mm2
......................................................................aagcaccacaguauaccagggUau..................   1     1   Mm2
......................................................................aagcaccacaguauaccagggcUu..................   5     1   Mm2
......................................................................aagcaccacaguauaccagggcau..................   5     0   Mm2
.......................................................................agcaccacaguauaccaCggc....................   1     1   Mm2
.......................................................................agcaccacaguauaccCgggc....................   1     1   Mm2
.......................................................................agcaccacaguauGccagggc....................   1     1   Mm2
.......................................................................agcaccacaguauaccagggc....................   196   0   Mm2
.......................................................................agcaccaAaguauaccagggc....................   1     1   Mm2
.......................................................................agcaccacaguGuaccagggc....................   1     1   Mm2
.......................................................................agcaccacaguauaccGgggc....................   1     1   Mm2
.......................................................................agGaccacaguauaccagggc....................   1     1   Mm2
.......................................................................Ngcaccacaguauaccagggc....................   1     1   Mm2
.......................................................................agcaccacaguauaccagggca...................   2     0   Mm2
.......................................................................agcaccacaguauaccagggcU...................   6     1   Mm2
........................................................................gcaccacGguauaccagggc....................   1     1   Mm2
........................................................................gcaccacaguauaccagggc....................   2     0   Mm2
.........................................................................caccacaguauaccagggc....................   5     0   Mm2
..........................................................................accacaguauaccagggc....................   1     0   Mm2

...................................ccugguauccagugguugcucuugg....................................................   9     0   Mm1
......................................................................aagcaccacaguauacAaggg.....................   2     1   Mm1
......................................................................aagcaccacaguauaccaggg.....................   169   0   Mm1
......................................................................aagcaccacaguauaccaggC.....................   1     1   Mm1
......................................................................aagcaccacaguauaccCggg.....................   1     1   Mm1
......................................................................aagcacAacaguauaccaggg.....................   1     1   Mm1
......................................................................aagcaccacaCuauaccaggg.....................   1     1   Mm1
......................................................................aagcaccacagGauaccagggc....................   1     1   Mm1
......................................................................aagcGccacaguauaccagggc....................   1     1   Mm1
......................................................................aagcaccacaguauaccagCgc....................   2     1   Mm1
......................................................................aagcaccacaguaGaccagggc....................   1     1   Mm1
......................................................................aagcaccacaguauaccaCggc....................   1     1   Mm1
......................................................................Cagcaccacaguauaccagggc....................   1     1   Mm1
......................................................................aCgcaccacaguauaccagggc....................   1     1   Mm1
......................................................................aagcaccaAaguauaccagggc....................   1     1   Mm1
......................................................................aagcaccacaguauaccaggUc....................   2     1   Mm1
......................................................................aagcCccacaguauaccagggc....................   2     1   Mm1
......................................................................aagcaccacaguauaUcagggc....................   1     1   Mm1
......................................................................aagcaccacaguaCaccagggc....................   1     1   Mm1
......................................................................aaAcaccacaguauaccagggc....................   3     1   Mm1
......................................................................aagcaccacaguaAaccagggc....................   1     1   Mm1
......................................................................aagcaccacaguauaccagggA....................   1     1   Mm1
......................................................................aagcaccacaguauaccaggCc....................   2     1   Mm1
......................................................................aagcaccacaguauacUagggc....................   32    1   Mm1
......................................................................aagcaccacaguauaccaggAc....................   1     1   Mm1
......................................................................aGgcaccacaguauaccagggc....................   3     1   Mm1
......................................................................aagcaccacagAauaccagggc....................   2     1   Mm1
......................................................................aagcaccacaguauaccagggU....................   4     1   Mm1
......................................................................aagAaccacaguauaccagggc....................   1     1   Mm1
......................................................................aagcaccCcaguauaccagggc....................   1     1   Mm1
......................................................................aagcaccacaguauaAcagggc....................   3     1   Mm1
......................................................................aagcaccacaguauacAagggc....................   42    1   Mm1
......................................................................Uagcaccacaguauaccagggc....................   7     1   Mm1
```

```
                                   Star                               Mature
uaucagauaauggagaaaauugcauggcuuuguccccugguauccagugguugcucuuggggacaugaccaagcaccacaguauaccagggcauaugcaugcuucuagauuc
......................................................................aagcaccacaguauaccUgggc....................    36      1      Mm1
......................................................................aagcaccacaguauGccagggc....................    1       1      Mm1
......................................................................aagcaccacaguauaccagggc....................    11780   0      Mm1
......................................................................aagcaccacaguUuaccagggc....................    2       1      Mm1
......................................................................aaUcaccacaguauaccagggc....................    2       1      Mm1
......................................................................aagcacAacaguauaccagggc....................    2       1      Mm1
......................................................................aagcaccacaguauaccagUgc....................    6       1      Mm1
......................................................................aagcacNacaguauaccagggc....................    1       1      Mm1
......................................................................aagcaccacaguauacGagggc....................    23      1      Mm1
......................................................................aagcaccaUaguauaccagggc....................    5       1      Mm1
......................................................................aagcaccacaguauaccGgggc....................    36      1      Mm1
......................................................................aagcaccacaAuauaccagggc....................    20      1      Mm1
......................................................................aagcaccacaguauaccagggG....................    2       1      Mm1
......................................................................aagUaccacaguauaccagggc....................    1       1      Mm1
......................................................................aagcaccacaCuauaccagggc....................    8       1      Mm1
......................................................................aagcaccacaguauaccCgggc....................    42      1      Mm1
......................................................................aagcaGcacaguauaccagggc....................    1       1      Mm1
......................................................................aUgcaccacaguauaccagggc....................    2       1      Mm1
......................................................................aagcaccacaUuauaccagggc....................    7       1      Mm1
......................................................................aagcUccacaguauaccagggc....................    1       1      Mm1
......................................................................aagcaccacGguauaccagggc....................    10      1      Mm1
......................................................................aagcaccUcaguauaccagggc....................    1       1      Mm1
......................................................................aagcaNcacaguauaccagggc....................    1       1      Mm1
......................................................................aagcaccacaguauaccagAgc....................    3       1      Mm1
......................................................................Nagcaccacaguauaccagggc....................    6       1      Mm1
......................................................................Gagcaccacaguauaccagggc....................    1       1      Mm1
......................................................................aagcaccacaguauaccaAggc....................    1       1      Mm1
......................................................................aagcacUacaguauaccagggc....................    4       1      Mm1
......................................................................aagcaUcacaguauaccagggc....................    2       1      Mm1
......................................................................aagcaccacaguauaccagggca...................    11      0      Mm1
......................................................................aagcaccacaguauaccagggcU...................    83      1      Mm1
.......................................................................agcaccacaguauaccagggc....................    21      0      Mm1

...................................ccugguauccagugguugcucuug.....................................................    2       0      Fm1
...................................ccugguauccagugguugcucuuggggacaugacc..........................................    7       0      Fm1
.....................................................................caagcaccacaguauaccaggg.....................    3       0      Fm1
.....................................................................caagcaccacGguauaccaggg.....................    1       1      Fm1
......................................................................aagcaccacaguauUccagg......................    1       1      Fm1
......................................................................aagcaccacaguauaccagg......................    11      0      Fm1
......................................................................aagcaccacaguauaccaggC.....................    4       1      Fm1
......................................................................aagcaccacaguauaccaggg.....................    35      0      Fm1
......................................................................aagcaccacaguauaccaCggc....................    1       1      Fm1
......................................................................aagcaccacaguauaUcagggc....................    5       1      Fm1
......................................................................aagcaccacaguUuaccagggc....................    4       1      Fm1
......................................................................aagcaccacaguauaccagggG....................    2       1      Fm1
......................................................................aagcaccacaguauaccagggU....................    48      1      Fm1
......................................................................Uagcaccacaguauaccagggc....................    13      1      Fm1
......................................................................aagcaccacaguauaccagCgc....................    4       1      Fm1
......................................................................aagcaccacaUuauaccagggc....................    9       1      Fm1
......................................................................aagcaccacagCauaccagggc....................    2       1      Fm1
......................................................................aagcaccacaguauaccCgggc....................    62      1      Fm1
......................................................................aGgcaccacaguauaccagggc....................    2       1      Fm1
......................................................................aagcaccacaguauacAagggc....................    68      1      Fm1
......................................................................aagcaccacaguauaccagUgc....................    2       1      Fm1
......................................................................aagcaccacaguaGaccagggc....................    4       1      Fm1
......................................................................aagcaccacaguauaccagAgc....................    1       1      Fm1
......................................................................aagcaNcacaguauaccagggc....................    4       1      Fm1
......................................................................aagcUccacaguauaccagggc....................    2       1      Fm1
......................................................................aagcaccacUguauaccagggc....................    5       1      Fm1
......................................................................aagcaccacaguauaccagggc....................    19185   0      Fm1
......................................................................aagcaccacaguauaccaggUc....................    1       1      Fm1
......................................................................aaUcaccacaguauaccagggc....................    6       1      Fm1
......................................................................aagUaccacaguauaccagggc....................    2       1      Fm1
......................................................................aagcCccacaguauaccagggc....................    2       1      Fm1
......................................................................aagcaccacaguauacGagggc....................    32      1      Fm1
......................................................................aagcaccacaguauaccaUggc....................    7       1      Fm1
......................................................................aagcGccacaguauaccagggc....................    2       1      Fm1
......................................................................aagcaccacaguauacUagggc....................    55      1      Fm1
......................................................................aagcaccacaguauaccUgggc....................    62      1      Fm1
......................................................................Cagcaccacaguauaccagggc....................    2       1      Fm1
```

Star Mature

```
uaucagauaauggagaaaauugcauggcuuuguccccugguauccagugguugcucuggggacaugaccaagcaccacaguauaccagggcauaugcaugcuucuagauuc
.....................................................................aUgcaccacaguauaccagggc....................  2     1  Fm1
.....................................................................aagcaUcacaguauaccagggc....................  2     1  Fm1
.....................................................................aagcaccacCguauaccagggc....................  1     1  Fm1
.....................................................................aagcaccacGguauaccagggc....................  13    1  Fm1
.....................................................................aaAcaccacaguauaccagggc....................  4     1  Fm1
.....................................................................aagcaccacaguaAaccagggc....................  4     1  Fm1
.....................................................................aagcaccacaCuauaccagggc....................  11    1  Fm1
.....................................................................aagcaccGcaguauaccagggc....................  7     1  Fm1
.....................................................................aagcaccacaguauaccaggCc....................  2     1  Fm1
.....................................................................aagcaccacaAuauaccagggc....................  31    1  Fm1
.....................................................................aagcaccacaguauaccaggAc....................  2     1  Fm1
.....................................................................Nagcaccacaguauaccagggc....................  19    1  Fm1
.....................................................................aagcaccUcaguauaccagggc....................  2     1  Fm1
.....................................................................aagcaccacagAauaccagggc....................  2     1  Fm1
.....................................................................aagcaccacaguaCaccagggc....................  2     1  Fm1
.....................................................................aagcaccacaguauaccGgggc....................  55    1  Fm1
.....................................................................aagcaccacaguauaccagggA....................  4     1  Fm1
.....................................................................aCgcaccacaguauaccagggc....................  1     1  Fm1
.....................................................................aagcacAacaguauaccagggc....................  1     1  Fm1
.....................................................................aagAaccacaguauaccagggc....................  2     1  Fm1
.....................................................................aagcaccacaguauUccagggc....................  1     1  Fm1
.....................................................................aagcaccaUaguauaccagggc....................  11    1  Fm1
.....................................................................aagcaccacaguauGccagggc....................  1     1  Fm1
.....................................................................Gagcaccacaguauaccagggc....................  4     1  Fm1
.....................................................................aagcacUacaguauaccagggc....................  9     1  Fm1
.....................................................................aagcaccacaguauaccagggcG...................  7     1  Fm1
.....................................................................aagcaccacaguauacUagggca...................  1     1  Fm1
.....................................................................aagcaccacaguauaccGgggca...................  1     1  Fm1
.....................................................................aagcaccacaguauaccagggcC...................  7     1  Fm1
.....................................................................aagcaccacaUuauaccagggca...................  1     1  Fm1
.....................................................................aagcaccacaguauaccagggAa...................  4     1  Fm1
.....................................................................aagcaccacaguauaccagggcU...................  237   1  Fm1
.....................................................................aagcaccacaguauaccagggca...................  53    0  Fm1
.....................................................................aagcaccaGaguauaccagggcau..................  29    1  Fm1
.....................................................................aagcaccacaguauaccagggcUu..................  5     1  Fm1
.....................................................................aagcaccacaguauaccagggcCu..................  2     1  Fm1
.....................................................................aagcaccacaguauaccagggcau..................  5     0  Fm1
.....................................................................aagcaccaGaguauaccagggcaua.................  2     1  Fm1
.....................................................................aagcaccacaguauaccagggcauU.................  2     1  Fm1
......................................................................agcaccacaguauaccagggc....................  28    0  Fm1
......................................................................agcaccacaguauaccagggcU...................  2     1  Fm1

...................................ccugguauccagugguugcucugg....................................................  9     0  Fm2
....................................................................caagcaccacaguauaccagggc....................  3     0  Fm2
.....................................................................aagcaccacaguauaccagg......................  4     0  Fm2
.....................................................................aagcaccacaguauaccaggg.....................  9     0  Fm2
.....................................................................aagcaccaUaguauaccagggc....................  14    1  Fm2
.....................................................................aagcGccacaguauaccagggc....................  2     1  Fm2
.....................................................................aGgcaccacaguauaccagggc....................  2     1  Fm2
.....................................................................aagcaccacaguauaccagUgc....................  1     1  Fm2
.....................................................................aagcaccacaguaCaccagggc....................  4     1  Fm2
.....................................................................aagcaccacaguauaccaUggc....................  4     1  Fm2
.....................................................................aagcacGacaguauaccagggc....................  1     1  Fm2
.....................................................................aagcaccacaguauacUagggc....................  43    1  Fm2
.....................................................................aagcaNcacaguauaccagggc....................  3     1  Fm2
.....................................................................aagcaccacagAauaccagggc....................  1     1  Fm2
.....................................................................aagcaccacaCuauaccagggc....................  7     1  Fm2
.....................................................................aagcaccacaguauGccagggc....................  3     1  Fm2
.....................................................................aagcaccaAaguauaccagggc....................  1     1  Fm2
.....................................................................aagcaccacaguauaccagggA....................  1     1  Fm2
.....................................................................aagcaccacaguauaccagAgc....................  1     1  Fm2
.....................................................................aagcaccacaguaAaccagggc....................  1     1  Fm2
.....................................................................aagcaccacaguauaccagggc....................  9753  0  Fm2
.....................................................................aagcaccacaguauacAagggc....................  29    1  Fm2
.....................................................................Gagcaccacaguauaccagggc....................  1     1  Fm2
.....................................................................aagcaccacaguauaccUgggc....................  28    1  Fm2
.....................................................................aagcUccacaguauaccagggc....................  1     1  Fm2
.....................................................................aaAcaccacaguauaccagggc....................  3     1  Fm2
.....................................................................aCgcaccacaguauaccagggc....................  2     1  Fm2
.....................................................................aagGaccacaguauaccagggc....................  2     1  Fm2
```

Star Mature

```
uaucagauaauggagaaaauugcauggcuuuguccccugguauccagugguugcucuuggggacaugaccaagcaccacaguauaccagggcauaugcaugcuucuagauuc
......................................................................aagcaccacaAuauaccagggc....................  10  1  Fm2
......................................................................aagcaccacaguauaUcagggc....................  15  1  Fm2
......................................................................aagcaccacaguauUccagggc....................  1   1  Fm2
......................................................................aagcaccacGguauaccagggc....................  3   1  Fm2
......................................................................aagAaccacaguauaccagggc....................  2   1  Fm2
......................................................................aagcaccacaguauaccagggU....................  21  1  Fm2
......................................................................Uagcaccacaguauaccagggc....................  6   1  Fm2
......................................................................aagcaccacaguauacGagggc....................  14  1  Fm2
......................................................................aagcaccacCguauaccagggc....................  1   1  Fm2
......................................................................aagcaccacaguauaAcagggc....................  2   1  Fm2
......................................................................aagcaccacaUuauaccagggc....................  9   1  Fm2
......................................................................aagcacAacaguauaccagggc....................  1   1  Fm2
......................................................................aagcaccacaguauaccCgggc....................  32  1  Fm2
......................................................................aagcacUacaguauaccagggc....................  4   1  Fm2
......................................................................aagcaccacaguauaccaggAc....................  2   1  Fm2
......................................................................aaUcaccacaguauaccagggc....................  5   1  Fm2
......................................................................Nagcaccacaguauaccagggc....................  4   1  Fm2
......................................................................aagcaUcacaguauaccagggc....................  2   1  Fm2
......................................................................aagcaccacaguauaccGgggc....................  28  1  Fm2
......................................................................aagcaccacaguauaccaggUc....................  1   1  Fm2
......................................................................aagcaccacaguauaccagggG....................  1   1  Fm2
......................................................................aagcaccacaguauacAagggca...................  1   1  Fm2
......................................................................aagcaccacaguauaccagggca...................  31  0  Fm2
......................................................................aagcaccacaguauaccagggcU...................  59  1  Fm2
......................................................................aagcacUacaguauaccagggca...................  1   1  Fm2
......................................................................aagcaccacaguauaccagggcaA..................  20  1  Fm2
......................................................................aagcaccacaguauaccagggcCu..................  8   1  Fm2
......................................................................aagcaccaGaguauaccagggcau..................  11  1  Fm2
.......................................................................agcaccacaguauaccagggc....................  29  0  Fm2
```
